# Interventions for improved diabetes control and self-management among those experiencing homelessness: protocol for a mixed methods scoping review

**DOI:** 10.1186/s13643-019-1020-x

**Published:** 2019-04-22

**Authors:** David J. T. Campbell, Rachel B. Campbell, Carolyn Ziegler, Kerry A. McBrien, Stephen W. Hwang, Gillian L. Booth

**Affiliations:** 1 Centre for Urban Health Solutions, Li Ka Shing Knowledge Institute, St. Michael’s Hospital, 30 Bond Street, Toronto, ON M5B 1W8 Canada; 2 Department of Medicine, University of Calgary, Calgary, AB Canada; 3 Department of Family Medicine, University of Calgary, Calgary, AB Canada; 4 Department of Community Health Sciences, University of Calgary, Calgary, AB Canada; 5 Department of Medicine, University of Toronto, Toronto, ON Canada

**Keywords:** Diabetes mellitus, Access to care, Homeless, Underserved, Marginalized, Health equity, Diabetes education, Self-management

## Abstract

**Background:**

Diabetes is a chronic medical condition that requires patients to be actively engaged in intensive self-management to achieve optimal clinical outcomes. Unfortunately, individuals who are experiencing homelessness often struggle to manage diabetes and consequently suffer numerous and severe complications—both acute and chronic. There are many barriers to optimal diabetes self-management among this population, and this may be exacerbated by the lack of tailoring and customization of care to this unique population. Given this disconnect, it is likely that many organizations have attempted to provide specialized innovations for this population—which may or may not be reported in the formal literature. Our objective is to perform a scoping review to summarize and synthesize the experiences of those who have attempted to provide tailored interventions.

**Methods:**

We propose a mixed methods scoping review that will include both a formal search of the published literature (MEDLINE, CINAHL, EMBASE, Web of Science, Scopus) and a thorough search of the grey literature. Eligible articles and documents are those that report on an intervention or guideline for the management of diabetes among those experiencing homelessness. All titles and abstracts will undergo duplicate review, as will the full article/document. We will include any report that either includes a description of an intervention or provides recommendations for the treatment of individuals who are homeless with diabetes. We will extract both qualitative and quantitative data for analysis and interpretation. Meta-analysis will not be performed.

**Discussion:**

Those experiencing homelessness who also have diabetes often struggle to manage their chronic condition. When care is tailored to suit their needs, it is feasible that outcomes may be improved. By collating and synthesizing information from diverse organizations and jurisdictions, we hope to facilitate the sharing of knowledge with others who wish to provide this type of care.

**Electronic supplementary material:**

The online version of this article (10.1186/s13643-019-1020-x) contains supplementary material, which is available to authorized users.

## Background

Individuals experiencing homelessness are often among the most underserved groups in society. Those faced with unstable housing often struggle to look after their mental and physical health [1, 2]. This presents unique challenges for those who are also affected by a chronic medical condition [3].

In order to achieve optimal health outcomes, chronic medical conditions, such as diabetes, require patients to engage in self-management [4, 5]. In order to avoid the debilitating complications that result from poor glycemic control, those with diabetes must not only adhere to numerous medical therapies, but also become effective self-managers. Diabetes self-management involves blood glucose monitoring [6] and subsequently adjusting therapies appropriately [7]. Additionally, health behavior changes are important, including following a specific diet [8], engaging in regular physical activity [9], and tobacco cessation [10].

Due to the various health and social challenges they face, patients with diabetes who also experience homelessness often have a difficult time with self-management [11]. They face financial barriers to accessing medications and testing supplies [3], food insecurity and lack of autonomy accessing nutritious foods [12, 13], social barriers and prejudice when seeking medical care [14, 15], and challenges storing medication and diabetic supplies (such as needles, insulin pens, testing strips, treatment for low blood sugar) [16]. Additionally, those experiencing homelessness often have problems with housing, employment, and/or mental health; in the face of these pressing priorities, their diabetes and physical health concerns may be neglected [17].

The result of these barriers is, in many cases, very poor glycemic control and, subsequently, adverse diabetes-related outcomes [11]. Those with type 1 diabetes are frequently in the hospital for potentially fatal acute diabetic emergencies such as severe low blood sugar [18] or diabetic ketoacidosis—caused by dangerously high blood sugar [19]. The need for hospital admissions due to suboptimal self-management and ambulatory care is substantially higher [20]. Chronic complications such as end-stage renal disease, myocardial infarction, and amputation are far more common in this group than in the general population [21].

In 2006, Glazier et al. published a review of diabetes interventions for “socially disadvantaged populations” [22]. The vast majority of the 17 studies reviewed by this group were in ethnic and racial minorities; none focused specifically on issues of those facing homelessness. More recently, a potentially more relevant manuscript was published by Hanlon et al. where they reviewed interventions for the homeless with the intent of improving “management of non-communicable diseases and communicable diseases requiring long-term care” [23]. This review reported predominantly studies of infectious diseases (tuberculosis, HIV, hepatitis) and found only one study focused on diabetes care. In this systematic review, the review of the grey literature did not include explicit search strategies for harder to find sources (such as blogs, webpages, reports, and meeting notes), sometimes termed “grey information” or “grey data” [24]. Furthermore, program descriptions and qualitative studies were excluded from this review. Given the limitations and different scope of these previous reviews, we feel there remains a need for our proposed review.

### Rationale

Given the high prevalence of diabetes-related complications experienced in this population, it has been postulated that individuals experiencing homelessness may not be engaging with traditional healthcare services (primary care, specialty care, and diabetes education services) due to myriad barriers. In response to these problems, we hypothesize that medical centers and community-based organizations have likely developed and piloted tailored practices, interventions, and novel models of care to aid this population in improving their self-management capacity and ability. Given that many of these interventions may not be reported in the published literature, we have proposed a scoping review to find, summarize, and synthesize the findings from these various programs.

### Purpose and objective

The objective of our scoping review is to find and synthesize any reports of tailored programs (or practice recommendations) for those with diabetes who have experiences with homelessness, to better understand what program models have been utilized and, where reported, to document the successes they may have achieved.

## Methods

We have envisioned a mixed methods scoping review. The objective of a mixed methods review has been described as: “combining the findings of qualitative and quantitative studies within a single systematic review to address the same overlapping or complementary review questions” [25]. This review has not been registered on PROSPERO, as scoping reviews are not eligible for registration. This protocol follows the format of the PRISMA-P checklist, and this review will be reported according to the guidelines recommended in the PRISMA Extension for Scoping Reviews checklist [26].

As many of the papers in this area may be process evaluations, qualitative studies, or simply program descriptions, we feel that it is important to conduct a mixed methods review, and will include both qualitative and quantitative data from the included reports. We will extract quantitative data from each study; however, due to the anticipated heterogeneity, we do not intend to pool or meta-analyze the results.

Scoping review methodology has been extensively described by Arksey and O’Malley [27] and further refined by Levac et al. [28]. One of the principal purposes of conducting a scoping review is:

To summarize and disseminate research findings: this kind of scoping study might describe in more detail the findings and range of research in particular areas of study, thereby providing a mechanism for summarizing and disseminating research findings to policy makers, practitioners and consumers who might otherwise lack time or resources to undertake such work themselves [27]

Scoping reviews often take a systematic approach to summarizing the literature in a given area. They differ, however, from a systematic review in that they do not attempt to limit their included studies by design or quality. Additionally, they generally do not have a quality appraisal component.

Arksey and O’Malley describe a five-stage process for undertaking a scoping review, which we will follow:

### Stage 1—Identifying the research question

We have attempted to create a research question which combines “a broad research question with a clearly articulated scope of inquiry,” as recommended by Levac et al. [28]. They recommend that prior to a scoping review, authors identify the following constructs of interest:

- Concept: Studies that identify specific interventions or guidelines/recommendations for tailored diabetes care for individuals experiencing homelessness
- Population: Individuals who are currently or have recently experienced homelessness
- Outcomes of interest (not required): Any diabetes/health/well-being-related outcomes, such as:
  - A1c
  - Diabetes complications
  - Hospitalizations
  - Diabetes distress
  - Quality of life
  - Mortality

### Stage 2—Identifying relevant studies

We will systematically search the published literature, in a similar fashion to a systematic review, but with no limits for study design. The search strategy was designed in conjunction with an experienced librarian (CZ), who is a member of the research team.

The databases we plan to search include MEDLINE, CINAHL, EMBASE, Web of Science, Scopus, and PsycInfo. Our search terms will include those for homelessness combined with the AND Boolean operator with terms for diabetes (see Additional file 1). We will thoroughly search through the reference lists from the manuscripts identified by the primary search.

In addition to the search of the formally published literature, we will undertake a thorough search of the grey literature, as we suspect that numerous programs and organizations may not have published any findings in the formal literature. This search will include targeted searches of dissertations/theses (ProQuest Dissertations & Theses Global) and conference abstracts (EMBASE Conference Abstracts, Conference Proceedings Citation Index—Science and Social Science & Humanities). We will broadly search using an Internet search engine (Google) and will perform targeted searches of relevant diabetes and homelessness agencies (see Additional file 2). Finally, for any programs/interventions discovered through the formal literature search, we will perform targeted Internet searching for additional/related documents.

### Stage 3—Study selection

We will undertake a typical two-stage screening process, as recommended by Levac et al. [28]: initially, titles and abstracts will be independently screened by two reviewers for relevance to the study question. In order to be as sensitive as possible, any article for which there is reviewer disagreement will automatically be advanced to the next stage of screening (full-text). Reviewer agreement will be calculated using the kappa statistic. At this stage, reports will only be excluded for the following reasons:

- Does not describe any intervention, nor provide recommendations
- Intervention clearly not specific to homeless populations (e.g., intervention for immigrants)
- Intervention clearly not relevant to diabetes, chronic disease management or primary health care

Reports that are identified in the initial screening stage will go to in-depth full-text review, which will also be conducted independently by two reviewers. Discrepancies will be resolved through discussion with a third party who will provide the deciding vote, should disagreement persist. The inclusion and exclusion criteria for full-text review are as follows:

#### Inclusion criteria

All reports or studies of an intervention designed to provide diabetes care to populations who are homeless or recently homeless—defined as:

1. Currently absolutely homeless (living in shelters or on the streets)
2. Currently in unstable housing situations (couch surfing, transiently housed)
3. Recently in one of the above situations (within the previous 5 years)

#### Exclusion criteria

4. No full text available (abstract only—however, we will attempt to contact authors to obtain full text of such studies).
5. No English, French, German, or Spanish language version available.
6. No intervention described, nor recommendations provided.
7. Intervention not specific to homeless populations (e.g., intervention for immigrants).
8. Intervention does not include aspects related to diabetes (e.g., mental health intervention). Note that care delivery interventions will be included if there is either a diabetes-specific focus or diabetes-related outcomes reported.
9. Intervention specifically for the prevention of diabetes, as opposed to treatment or management of pre-existing diabetes.

We will not exclude any manuscript or report on the basis of study design. Included study types and reports will include quantitative studies (randomized trials and observational studies), qualitative studies (qualitative description, grounded theory, etc.), and program evaluations as well as program descriptions, provided they meet the criteria above. There are no limitations placed on specific outcomes, study quality, risk of bias, location, or timeframe.

References will be managed with EndNote Web software throughout the review process. Colandr will be used for study selection and review processes.

### Stage 4—Charting the data

Once the final set of studies is chosen, data abstraction will be completed. Initially, we will chart details about the individual studies and programs, including year, location, target populations, and intervention components.

As this is a mixed methods review, both quantitative and qualitative data will be abstracted. Quantitative data and statistics will be extracted, including types of interventions, outcomes evaluated, results, and statistical significance. Qualitative data will be extracted for meta-ethnographic analysis. The entire results and discussion sections of relevant qualitative papers will be copied and pasted directly into a separate document for textual analysis.

### Stage 5—Collating, summarizing, and reporting results

Quantitative data will be presented in summary tables. If there are sufficient numbers of studies, we may group the individual studies by intervention type (i.e., diabetes education, dietary intervention, medication and diabetes supply subsidies).

Qualitative data will be imported into NVivo 12 software (Doncaster, Australia) for analysis. We intend to conduct a detailed thematic analysis on this data [29]. Open coding will proceed in a line-by-line fashion that will make use of a preliminary coding template (Table 1). Codes will be added inductively throughout the process of data analysis. Focused coding will be achieved by thoroughly reviewing codes and grouping them into similar groups that will be abstracted to themes.

**Table 1:** Preliminary coding template

Parent node	Child node	
Program description	Dietary intervention	
	Tailored diabetes education	
	Facilitated medication access	
	Facilitated supply access	
	Social worker/housing	
	Group support	
Program characteristics	Convenient location	
	Convenient hours of operation	
	Social support	
	Provider	MD primary care
		MD specialist
		RN
		RD
		Pharmacist
Patient benefits	Improved engagement	
	Improved process markers (labs, screening)	
	Improved outcomes (clinical)	
	Improved outcomes (patient-reported)	
Staff and system benefits	Improved satisfaction	
	Decreased workload	
	Decreased resource utilization (i.e., acute care)	
Challenges	Lack of engagement	
	No show	
	Cost of running program	
	Increased workload	

## Discussion

Individuals who are experiencing homelessness are known to have a higher incidence of diabetes-related adverse outcomes. There are many contributing factors to this disparity, including poorer access to medical care, financial barriers to medications and supplies, lack of trust with healthcare providers, and competing priorities. Additionally, a significant contributor to the disparities in outcomes is likely related to the fact that diabetes care is often not tailored to the specific needs and challenges of those experiencing homelessness.

### Limitations

Given the significant heterogeneity in the types of interventions and the outcomes reported, we are not anticipating being able to meta-analyze the results of our review. Therefore, in all likelihood, we will be unable to provide any insight into the most helpful or successful aspects of various programs. However, we feel that our review will be valuable, providing direction for future research and practice by sharing information about the types of programs that have been implemented.

The success and usefulness of this review is fully contingent upon organizations producing some form of written record or report documenting their experiences that are able to be found through online searches. It is entirely possible that many organizations will not have such documentation available. In such cases, we will be unable to include these programs in our report.

## Conclusion and implications

Undoubtedly, organizations dedicated to improving diabetes care, and those whose mandate is to care for those experiencing homelessness, have noticed the significant care gap for this population, and have devised programs to address this problem. However, since much of this innovative work is likely happening on the ground in community-based organizations and individual practices, knowledge of novel programs and innovations is less likely to be disseminated across regional jurisdictions. The purpose of this review is to gather, synthesize, and disseminate the learnings across organizations. It is our hope that those considering implementing programs or new approaches to diabetes care for individuals experiencing homelessness will be able to reference our review as they plan their services to profit from the learnings of others.

## Additional files

Additional file 1: MEDLINE search terms (DOCX 14 kb)

Additional file 2: Grey literature internet searching terms (DOCX 16 kb)

## Abbreviations

A1c: Glycosylated hemoglobin
HIV: Human immunodeficiency virus
PRISMA: Preferred Reporting Items for Systematic Reviews and Meta-Analyses
PROSPERO: International Prospective Register of Systematic Reviews
