# Supplementary material for: Interventions for improved diabetes control and self-management among those experiencing homelessness: protocol for a mixed methods scoping review
Source: Syst Rev. 2019 Apr 22;8:100. doi: 10.1186/s13643-019-1020-x (PMC6477731; doi:10.1186/s13643-019-1020-x)
Supplement: Supplementary file 1 — MEDLINE search terms (DOCX 14 kb) [file 13643_2019_1020_MOESM1_ESM.docx]

**Appendix A: Medline Search Terms**

Database: Ovid MEDLINE: Epub Ahead of Print, In-Process & Other Non-Indexed Citations, Ovid MEDLINE® Daily and Ovid MEDLINE® <1946-Present>

Search Strategy:

--------------------------------------------------------------------------------

1     exp Homeless Persons/

2     Emergency Shelter/

3     homeless*.tw,kf.

4     no fixed address.tw,kf.

5     underhoused.tw,kf.

6     roofless.tw,kf.

7     seeking shelter.tw,kf.

8     unhoused.tw,kf.

9     street involved.tw,kf.

10     sleeping rough.tw,kf.

11     unstabl* hous*.tw,kf.

12     housing instability.tw,kf.

13     precarious* hous*.tw,kf.

14     vulnerably housed.tw,kf.

15     emergency shelter*.tw,kf.

16     housing first.tw,kf.

17     transitional shelter*.tw,kf.

18     marginally housed.tw,kf.

19     skid row.tw,kf.

20     couch surf*.tw,kf.

21     Emergency Shelter/

22     street youth*.tw,kf.

23     street people.tw,kf.

24     living on the street*.tw,kf.

25 doubled up.tw,kf

26 transitiona* hous*.tw,kf

27 support* hous*.tw,kf

28 housing first.tw,kf

29     or/1-28

30     exp Diabetes Mellitus/

31     Diet, Diabetic/

32     diabet*.tw,kf.

33     30 or 31 or 32

34     29 and 33

***************************

  /     Medical Subject Heading (MeSH)

exp   means that there are narrower MeSH headings underneath

*       truncation (e.g. child* finds child or children or childhood etc.)

.tw.  textword (word/phrase appears in title or abstract)

.kf.  keyword field  (author assigned keywords)

(###) number of results
