# Supplementary material for: Interventions for improved diabetes control and self-management among those experiencing homelessness: protocol for a mixed methods scoping review
Source: Syst Rev. 2019 Apr 22;8:100. doi: 10.1186/s13643-019-1020-x (PMC6477731; doi:10.1186/s13643-019-1020-x)
Supplement: Supplementary file 2 — Grey literature internet searching terms (DOCX 16 kb) [file 13643_2019_1020_MOESM2_ESM.docx]

**Appendix B: Grey Literature Internet Searching Terms**

Targeted Searching:

We will search for “diabetes” on the following homelessness sites:

- Canadian Observatory on Homelessness/Homelesshub.ca (CAN);
- Canadian Alliance to End Homelessness (CAN);
- National Coalition for the Homeless (USA);
- National Alliance to End Homelessness (USA);
- National Health Care for the Homeless Council (USA);
- European Observatory on Homelessness (Europe);
- Homeless Link (UK); and
- Homelessness Australia (AUS)

We will also search “homeless” and “homelessness” on the following diabetes sites:

- Diabetes Canada;
- American Diabetes Association;
- European Association for the Study of Diabetes;
- Diabetes UK;
- Diabetes Australia;
- Diabetes New Zealand;
- Diabetes Ireland;
- Diabetes South Africa; and
- International Diabetes Federation

Generic Searching:

For each of the following generic google search terms we will screen 20 pages for potentially relevant entries:

“Homeless” AND “Diabetes”

“Homeless” AND “Diabetic”

“Homelessness” AND “Diabetes”

“Homelessness” AND “Diabetic”

We will then specify by city, and screen 5 pages of google entries for each:

“Homeless” AND “Diabetes” AND “CITY NAME”

We will search the most populous cities in predominantly English speaking countries:

- USA (50 municipalities)
- Canada (10 municipalities)
- UK (10 municipalities)
- Ireland (3 municipalities)
- Australia (10 Municipalities)
- South Africa (8 Municipalities)
- New Zealand (4 municipalities)

US Cities:

1. New York, NY
2. Los Angeles, CA
3. Chicago, IL
4. Houston, TX
5. Phoenix, AZ
6. Philadelphia, PA
7. San Antonio, TX
8. San Diego, CA
9. Dallas, TX
10. San Jose, CA
11. Austin, TX
12. Jacksonville, FL
13. San Francisco, CA
14. Columbus, OH
15. Fort Worth, TX
16. Indianapolis, IN
17. Charlotte, NC
18. Seattle, WA
19. Denver, CO
20. Washington, DC
21. Boston, MA
22. El Paso, TX
23. Detroit, MI
24. Nashville, TN
25. Memphis, TN
26. Portland, OR
27. Oklahoma City, OK
28. Las Vegas, NV
29. Louisville, KY
30. Baltimore, MD
31. Milwaukee, WI
32. Albuquerque, NM
33. Tucson, AZ
34. Fresno, CA
35. Sacramento, CA
36. Kansas City, MO
37. Atlanta, GA
38. Long Beach, CA
39. Omaha, NB
40. Raleigh, NC
41. Miami, FL
42. Oakland, CA
43. Minneapolis, MN
44. Tulsa, OK
45. New Orleans, LA
46. Cleveland, OH
47. Tampa, FL
48. Honolulu, HI
49. St. Louis, MO
50. Pittsburgh, PA

Canadian Cities:

1. Toronto, ON
2. Montreal, QC
3. Vancouver, BC
4. Calgary, AB
5. Edmonton, AB
6. Ottawa, ON
7. Winnipeg, MB
8. Hamilton, ON
9. Regina, SK
10. Halifax, NS

UK cities

1. London
2. Birmingham
3. Liverpool
4. Leeds
5. Sheffield
6. Bristol
7. Manchester
8. Glasgow
9. Edinburgh
10. Belfast

Irish cities

1. Dublin
2. Cork
3. Limerick

Australian Cities

1. Sydney
2. Melbourne
3. Brisbane
4. Perth
5. Adelaide
6. Gold Coast
7. Newcastle
8. Canberra
9. Sunshine Coast
10. Hobart

South African Cities

1. East London
2. Cape Town
3. Johannesburg
4. Pretoria
5. Germiston
6. Durban
7. Bloemfontein
8. Port Elizabeth

New Zealand Cities

1. Auckland
2. Wellington
3. Christchurch
4. Hamilton
